# Registered nurses in Israel - workforce employment characteristics and projected supply

**DOI:** 10.1186/2045-4015-1-11

**Published:** 2012-03-12

**Authors:** Nurit Nirel, Shoshana Riba, Sima Reicher, Orly Toren

**Affiliations:** 1 Smokler Center for Health Policy Research, Myers-JDC-Brookdale Institute, JDC Hill, POB 3886, Jerusalem 91037, Israel; 2 Nursing Division, Ministry of Health, Ben-Tabai 2, Jerusalem 93591, Israel; 3 Hadassah University Medical Center, Jerusalem, 91120, Israel

## Abstract

**Background:**

Surveys of nursing supplies around the world have furnished a better understanding of the structure of the workforce, helped identify shortages, and plan professional training. This study aimed to examine the employment and workforce characteristics of registered nurses and the projected supply in Israel as a tool for planning.

**Methods:**

1. A survey of a national sample of 10% of the RNs of working age (3,200 nurses). 2. Analysis of administrative data from the Ministry of Health' Nursing Division and the Central Bureau of Statistics.

**Results:**

Most registered nurses are employed (89%) - 67% work full time. The workforce is mature (45% are above 45), trained (55% qualified beyond the basic course, 48% hold a BA, 18% hold an MA or PhD), and stable: few quit the profession altogether. The likelihood of "survival" in the profession after 10 years is 93%; after 20 years - 88%. 23% have made some transition in the last 10 years (most - a single transition). Most of the transitions are from hospital to community work. Supply projections show a decrease in the total number of RNs in the nursing workforce from 28,500 in 2008 to 21,201 in 2028 - i.e., of 25% by the end of the period. As for the ratio per 1,000 population, the drop is from 4 registered nurses/1,000 in 2008 to 2/1,000 in 2028.

**Conclusions:**

The study findings provide more rigorous projections of supply than in the past on the declining rates of the nursing workforce in the coming decades, and contribute to decision making about the scope of training and recruitment. The study also points to the implications for policy decisions regarding the findings that the young nursing workforce is less stable, that there are advantages to recruiting a more mature workforce, and that post-basic education is connected with workforce stability.

## Background

Western healthcare systems suffer from a severe nursing shortage and imbalance between workforce demand and supplies [1]. In Israel in the past two decades, two main trends have impacted nursing supply: (a) the entry of thousands of immigrant nurses into the profession as practical nurses, and the policy of the Ministry of Health (MoH) to retrain as RNs the practical nurses licensed in Israel, and (b) the fact that practical nurses stopped being trained (since 2007) and registered as licensed nurses (since September 2009) in accordance with the recommendations of the Nursing Manpower Planning Committee [2]. This is in keeping with a policy of workforce development aimed to training advanced, academic RNs able to bring higher knowledge and skills to patient care. In the past, opinion was divided over the question of whether Israel faced an impending nursing shortage. Today, however, the parties concerned appear to be in general agreement that such a shortage indeed exists.

Surveys of nursing supplies in the developed world have furnished a better understanding of the workforce structure, helped identify shortages, and plan professional training [3,4]. Israel's MoH keeps records of the number and distribution of nurses by clinical specialty (post basic education) and professional status while the Central Bureau of Statistics (CBS) publishes estimates of employed nurses by geographic location. Yet, as a planning aid, there has been no comprehensive survey of nurses' employment characteristics and distribution by clinical field or economic sector, the percentage leaving work and the lifespan of full- or half-time work. This has hampered the assessment of the existing nursing workforce against future supplements, information that would facilitate decision-making and workforce planning.

Planning workforce supply entails an examination of the employed (active) and unemployed (inactive) workforce, and projected entries and exits (including retirement, death or emigration). Planning is based on projections of exit/entry balances [5,6]. If imbalance derives from distribution, it may not suffice to examine the general nursing supply. Uneven distribution may apply to nursing specialties [7], institutions and services, public and private sectors, geographic localities [7-12] or professional mobility. Workforce supply examinations will thus consider various balances (e.g., service or geographic differences) and internal/external mobility trends (within nursing versus leaving the profession) [13]. The construction of entry/exit models for workforce projections requires high-quality data for reliable evaluations [8].

This study aimed to provide an in-depth review of the existing supply of Israel's nursing workforce: job and employment characteristics, internal and external mobility, working lifespan and the projected RN supply in view of exits and future entry sources.

## Methods

1. A random sample of licensed RNs of working age (whether employed or not) were interviewed via telephone from October 2008 to February 2009, using a closed questionnaire.

2. Analysis of the following annual administrative data:

a. New recipients of RN licenses

b. Graduates of post basic clinical courses, by specialty

c. Nurse emigration -MoH Nursing Division data

d. Mortality - CBS data

### Study Population

The study population numbered 32,000 RNs of working age (up to 60). Practical nurses were not included, as the future workforce will consist of RNs only (see introduction) ^1^.

### Sampling Framework, Methods and Size

A simple, random sample was drawn from the MoH file of licensed nurses: 4,500 names from which 10% of working-age RNs were to be interviewed. The sample size was calculated on a presumed 70% response rate based on previous experience (dropout, change of surname [due to marriage], of address etc. A preliminary letter was sent to entire sample explaining the purpose and importance of the study along with assurances of maintaining confidentiality, and an enclosed return envelope. Those who refused to be interviewed were asked to advise of same by return mail, email or telephone and were not approached by the researchers. We interviewed 3,200 RNs, with a response rate of 72%; 13% (570 nurses) were not located; 7% (318) refused to be interviewed; an additional 2% lived overseas and were unreachable, and 6% were not interviewed due to the survey's termination.

### Study Tools

The questionnaire variables were demographics; completion date of basic qualification; type and date of completed advance training; current employment; organizational position/level; professional seniority. Employment histories helped gauge internal mobility: nursing entries/exits, transfers between economic sectors or hospital and community, within hospital and community work, and changes in full- or part-time work. Employment characteristics examined the extent of daily/weekly hours, of shift work and shift flexibility. Before the start of the survey, the questionnaire was checked by a pre-test to verify that it was clear and reliable.

### Statistical Analyses

The Chi^2 ^test was used to examine the interdependence of non-quantitative variables (measured on a nominal scale); the T test - for the significance of differing averages; and multivariate analysis (logistic regression) with the T test to check the variables' independent impact. The Kaplan-Meier survival analysis measured the liklihood of RNs remaining in the profession after X years. The cumulative survival likelihood calculated for given periods was used to assess the overall probability of quitting work in the profession after specific lengths of time. As RN cohorts were examined retrospectively, (according to their entry into the profession), the study employed historical prospective analysis. The model was based on the following formula:

$$Ŝ\left(t\right)=\underset{t_{i}<t}{\prod }\frac{n_{i}-d_{i}}{n_{i}}.$$

When:

S is the proportion of respondents surviving in the profession beyond follow-up (t)

n_i _is the number of interviewees before t_i_

d_i _is the number of interviewees who dropped out of the profession before t_i_

Our assumption was that with regard to employment histories, we would not be able to obtain reliable informative data for periods going back more than 10 years; we therefore asked about the past 10 years only in questions examining these topics (employment histories).

To examine the factors predicting RN exits from work in the nursing profession, we used a Cox multivariate regression analysis. In this type of regression analysis, the dependent variable - the likelihood of leaving work in the profession - has two components: time (duration of work as an RN) and "occurrence" (quitting or continuing work in the profession); i.e., the likelihood of leaving work in the profession after a given number of years. The independent variables in this analysis were: age, gender, family status, place of birth, post-basic nursing training, academic education, economic sector, community vs. hospital work, place of residence.

The supply projections were drawn up with the help of Excel tables, according to the following model:

Currently employed workforce **+ **entry into workforce (addition of new RNs; addition of practical nurses retrained as RNs; returning nurses, over 5 years) **- **exits from workforce (retirement and dropout; deaths over 5 years) **= **projected supply

## Results

### 

#### Potential Workforce Entries

Potential future RN sources consist of the graduates of academic nursing tracks in colleges and universities, of three-year diploma programs, of second-career courses and of practical nurses to qualify as RNs. In 2000-08, an annual average of 1,600 new RN licensees^2 ^entered the workforce; in 2009 - 1,218. In 2008, 56% of the new RNs were younger than 30, 37% were aged 30-44, and 7% were over 45 [14,15].

Formerly, nurses training applied also to practical-nursing tracks, which were considerably downsized recently and since 2007 are no longer available. Indeed, the percentage of RNs among licensed nurses under 60 rose from 63% in 2000 to 78% in 2008 [14], a trend expected to continue in the coming years.

The immigration wave from the former Soviet Union (FSU) starting in 1990 imported many nurses who joined the workforce as practical nurses. MoH policy was to "upgrade" them, allowing them to improve their qualifications in RN retraining courses. In 2000-08, an annual average of 600 practical nurses joined the RN workforce after retraining. In 2009, the number fell to 380 as the FSU influx ended. Presumably, those practical nurses able to requalify as RNs had already done so and, as said, since 2007 practical nurses are no longer being trained. The reservoir of practical nurses is thus "drying up." One may assume that in the five-year period of 2008-2013 the amount of practical nurses requalifying as RNs will be halved to an annual 300, and in the following five-year period - even further, to an annual 150: a quarter of the amount of nurses requalifying in 2000-08. Ten years later, in 2028, there are expected to be very few practical nurses requalifying as RNs.

At the same time, nurses training has expanded somewhat with a commitment from the Finance Ministry to fund more second-career and academic programs and increase the number of students in nursing college (for another 2100 students). MoH data show that the number of students in second-career courses rose by 133%, from 193 in 2008 to 450 in 2009 [15] and, in nursing college, by 33%, from 186 to 219 [14,15]. The number of college students will presumably grow in the coming years especially as an additional nursing college is planned. In other words, an increase is expected in the number of new RNs completing academic retraining from 2012 and those graduating from academic college/university nursing tracks from 2013. Thus, in the 2009-2013 five-year period, an average annual addition of 1,130 newly-licensed RNs is expected - totaling 5,650.

### Existing Workforce

#### Workforce Characteristics

The data show the current nursing workforce to be mature - 45% are 45 and older; 90% are women and 56% were born in Israel. About 40% work in the center of the country, 27% in Haifa and the north, 22% in the south (though only14% live there), 11% in Jerusalem and 1% in the West Bank. Community RNs fall into the 45-61 age group (50%) more so than hospital RNs (42%); they have a higher proportion of women (92%) and Israeli born (64%) than hospital nurses (52% and 88% respectively), and a higher proportion work in Haifa and the north, the south, and the West Bank (52%) than hospital nurses (40%). The current workforce was found to be trained and skilled: 55% of RNs have advance training, 48% hold an undergraduate degree, and 18% hold an MA or PhD.

#### Employment Characteristics

Most RNs (89%) do work in nursing; of these, 74% work in hospitals and 26% in the community. Most (90%) work at a single job. The rate of full-time employees is 67% and, predictably, higher among hospital nurses (73%) than community nurses (52%). A nurse works an average of 37.9 hours per week: hospital nurses work on average 38.5 hours (more than the 36 hours required by labor agreements); community nurses work on average 36.5 (less than the 40 hours required by labor agreements). According to the findings, 41% work in the government/municipal sector; 36% - in health plans; and the remainder in the public (13%) or private sector (10%).

Furthermore, the current workforce is stable; few quit working in the profession. The percentage of RNs in the sample that stopped working as nurses in the past decade is very low - less than 1% per year. In that same period, 23% of today's employed RNs made some sort of transition/s within the profession, whether between sectors, types of hospital/community units, from community to hospital or vice versa (60% made a single transition). Most of the transitions were from hospital to community work. Another aspect of nursing stability relates to temporary leaves and re-entries. When we asked employed nurses at the time of the survey: "In the past 10 years, did you stop working as a nurse for a period of more than six months?", 13% reported that they had. Of these, 82% reported one period of leave, the rest reported two or more. The average number of leaves/re-entries was 1.4. Most nurses (58%) reported that their latest period of leave had lasted a year, 24% reported a period of six to 12 months, and 16% reported leave of at least two years. Based on these data, we estimated the average annual percent of nurses on temporary leave from work in the profession: the number of nurses responding affirmatively was multiplied by 10% (the "value" of each year) and by 1.4 (the average number of nursing exits and entries in the entire sample). This calculation showed an annual average of 1.86% for nurses leaving/re-entering the workforce, the percentage varying with age group (Table 1).

**Table 1:** Nurses outside of the nursing workforce for a period of 1 year in the past 10 years, by age

Age group N = 2856	Total^1^ n = 2841	24-29 n = 205	30-34 n = 435	35-39 n = 496	40-44 n = 451	45-49 n = 416	50-54 n = 371	55-60 n = 467
**Percentage of nurses reporting leave of at least 6 months in each age group**	**13.3**	12.2	15.9	20.4	18.0	10.8	7.8	6.0

**Percentage of age group temporarily outside of the workforce**	**1.9**	1.7	2.2	2.9	2.5	1.5	1.1	0.8

^1 ^This table is based on data on all the nurses working in the profession today (**n **= 2,856). As the age of 14 respondents was unknown, there is a discrepancy between the expected **n **and the number of respondents on the two questions.

#### The Likelihood of New RNs Remaining to Work in the Profession

**Figure 1:**
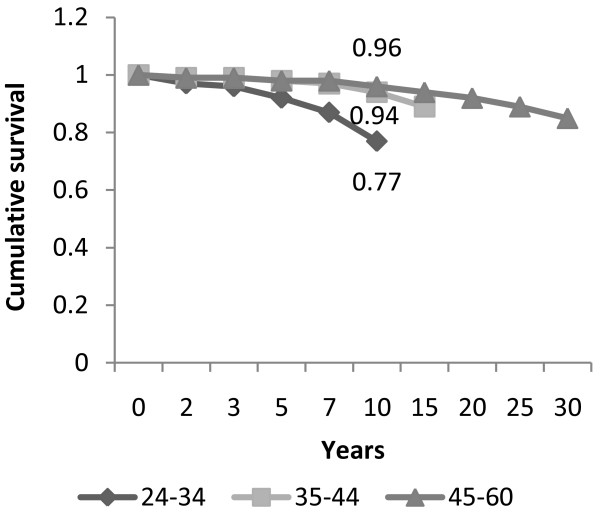
**Cumulative survival in the nursing profession, by age group**.

**Figure 2:**
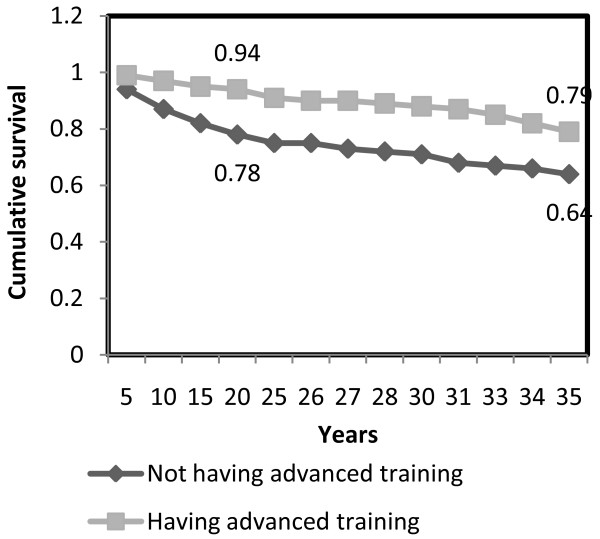
**Cumulative survival in the nursing profession, by presence/absence of advance training**.

An examination of the cumulative survival rate of working RNs (based on the data on all employed or formerly employed nurses) showed a 97% likelihood that RNs who had started working in the profession would continue to do so after five years. After 10 years, the survival rate was 93% and after 20 years - 88%. The survival rate varied with age group: a comparison by age group of the current workforce showed a 77% likelihood of young nurses remaining in the profession after 10 years versus 94% and 99% for older age groups. Moreover, after 10 years, the survival rate of nurses with post-basic education was 97% versus 87% for nurses with only basic training; after 20 years - the survival rates were 94% versus 78% respectively (Figures 1 and 2). In addition, the cumulative survival after 10 years of seniority in the profession was 96% in the group of RNs who began to work before 1998, compared with 87% among nurses who began to work in the past decade (since 1998).

#### Variables Predicting Exits from Work in the Nursing Profession

To examine the factors predicting RN exits from work in the nursing profession, we used a Cox multivariate regression analysis. After controlling for the different variables in the regression equation, we found that age, post-basic education, economic sector and family status independently affected the likelihood of leaving. The likelihood was significantly higher in the 24-34 age group. The survival likelihood of the 24-34 age group was five time lower than that of the of 34-44 age group, and four times lower than in the 45-62 age group. Similarly, the likelihood of leaving work as a nurse was nearly three times higher among RNs with no advance training than among higher-qualified nurses; 1.6 times higher among RNs working in the private sector versus the government/public sector or health plans; and significantly lower among married RNs with no children under 18 than among those in other family situations.

#### Projected Nursing Supply

The projected RN supply was examined in view of the existing nursing workforce (the number of employed RNs up to age 60), the projected exits from work in the profession (including retirement, death or emigration) and future sources. Supply projections were made for a few points in time at five-year increments from the baseline of 2008 (the year the study was launched). The Cox regression analysis showed the age variable to impact exits from the profession. The age variable is related to rates of entry into the nursing profession as well as to mortality rates. The study findings made it possible to include this variable in the equations of projected supply since we possessed data on the distribution of employed nurses and their survival rates by age group. We also had estimates of age-group distribution in future sources of additional RNs and future exits from the nursing workforce.

Supplies were projected on the assumption that, apart from natural changes in age-group distribution, there would be a significant decrease in the numbers of retraining from practical to registered nurses, along with an increase in the extent of training of new RNs^3^. This is in keeping with the above mentioned sources of professional manpower whereas the profession's entry and exit rates, which are dependent on other variables, would remain the same as in the past. The projections rested on the following data:

1. The existing workforce - study findings on the proportion of RNs working in the profession at the time of the survey, by age group

2. Projected annual nursing entries - rates of annual entries into the nursing workforce, by age group, according to data of the MoH Nursing Division

3. Projected annual nursing exits - dropout data (including retirement) based on survival analysis of study data on remaining in the profession in the given period, by age group; projected workforce returns, based on the percentage of nurses annually leaving/returning to the workforce, by age group; estimated annual RN emigration rate of 0.0076, by age group, according to data of the MoH Nursing Division on the number of certification requests for employment abroad; and mortality rates, by age group, based on women's mortality rates/1,000, according to CBS data.

Table 2 shows the projected nursing supply in five years time, in 2013. It shows that the number of RNs (under 60) who work in nursing, which stood at 28,460 in 2008, will drop to 24,853 in 2013; a decline of some 12.5% over five years. Similarly, we projected the supply of RNs at five-year increments: 2018, 2023 and 2028. Table 3 presents the summarized projections for the nursing workforce at these points in time. It shows a downward trend in the projected totals of RNs in the workforce from 28,460 working in the profession in 2008 to 21,201 20 years later, in 2028; a drop of 25.5% by the end of the period; in terms of the ratio of working RNs/1000 population, a drop from 3.88/1,000 in 2008 to 2.18/1,000 in 2028 (Table 4). Note that the rate of licensed RNs under 60 per 1,000 population is 4.42 for 2008 and 4.43 in 2009 [15].

**Table 2:** Projected supply of nurses in 5 years time (2013), by age group

Age group	Distribution 2008^1^	After 5 Years^2^	Addition of New RNs^3^	Addition of Practical Nurses Retrained to RNs^4^	Retirement and Drop-out over 5 Years^5^	Emigration over 5 Years^6^	Deaths over 5 Years ^7^	Return to Nursing over 5 Years^8^	Projection 2013^9^
**Total**	**28,460**	**23,790**	**5,639**	**1,500**	**4,792**	**1,080**	**202**	**97**	**24,953**

24-29	2060	-	4018	420	1030	78	2	18	3345

30-34	4370	2060	461	295	1748	166	9	39	933

35-39	4960	4370	461	295	744	188	12	21	4203

40-44	4530	4960	461	295	227	172	20	6	5303

45-49	4160	4530	238	195	624	158	31	9	4159

50-54	3710	4160	-	-	186	141	41	2	3795

55-60	4670	3710	-	-	234	177	86	2	3215

^1 ^Number of RNs in the sample working during the survey (89%), was multiplied by 10 in each age group to reflect the distribution in the general population of employed RNs.

^2 ^Distribution by age of RNs working after 5 years (the entire group moved over to the next age group)

^3 ^Distribution of expected new RN entries, by age group (according to MoH data on the average annual number of new RNs)

^4 ^Distribution of expected entries of RNs retrained from practical nurses, by age group (according to MoH data on the average annual number of re-trainees from practical to registered nurses)

^5 ^Dropout rate (due to retirement or quitting the profession of RN) was calculated on the basis of cumulative survival analysis, by age group. The numbers were calculated as the product of the dropout rate times the number of nurses in the age group at the start of the period, and multiplied by 5 to reflect exits from nursing work over 5 years

^6 ^An emigration rate of 0.0076 in each of the age groups multiplied by 5 to reflect emigration over 5 years

^7 ^The product of the mortality rate/1,000 (women) in that age group (CBS data) times the number of RNs in the age group, multiplied by 5 for years and divided by 1,000

^8 ^Percentage of nurses likely to return to the profession in each age group (according to the distribution of this percentage, by age group as in Table 2), multiplied by the number of nurses in each age group

^9 ^The sum of the number of nurses at the end of the period, the amount of new entries and the amount of potential returnees (3^rd^, 4^th ^and 8^th ^columns) minus the sum of the columns presenting workforce exits - dropout, emigration, death (columns 5, 6 and 7)

**Table 3:** Projected supply of RNs at given times (absolute numbers)

Baseline Year	2008	2013	2018	2023
**Total RNs in baseline year**	**28460**	**24953**	**24413**	**22692**

After 5 years (without new additions)	23790	21738	20953	19259

Addition of new RNs over 5 years	5640	6587	6587	6587

Addition of practical-RN retrainees over 5 years	1500	750	375	190

Retirement and dropout over 5 years	4792	3915	4544	4210

Emigration over 5 years	1080	947	926	861

Deaths over 5 years	202	172	175	166

Returning to nursing workforce over 5 years	97	372	422	402

Year marking end of period	2013	2018	2023	2028

Projected nursing supply at end of period	24953	24413	22692	21201

**Table 4:** Projected supplies of RNs per 1,000 population at given times

Year	Nursing Supply	Population (in 1,000s)	Nurses/1,000
2008	28460	7343	3.88

2013	24953	8174.5^1^	3.15

2018	24413	8770^2^	2.86

2023	22692	9367.6^3^	2.49

2028	21201	9984.6^4^	2.18

Population forecasts are taken from Israel's projected population up to 2030, CBS (2009) - the medium variant.

The Central Bureau of Statistics (CBS) projects Israel's population growth every few years. The latest projections were published in 2008, based on population estimates at the end of 2005. The forecasted population growths in Table 4 are based on the projections for 2015, 2020, 2025, 2030 and interpolations for 2013, 2018, 2023, 2028, for which we have the projected number of RNs. Interpolation is geometric, according to an assumed percentage of steady growth in each 5-year increment.

^1 ^CBS projection 2015

^2 ^CBS projection 2020

^3 ^CBS projection 2025

^4 ^CBS projection 2030

Apart from natural changes in the age of employed RNs, the major projected drop in their numbers relates to the decrease in re-trainees from practical to registered nurses in another 10 or 20 years. Clearly if the extent of RN training expands, supply will change accordingly. Similarly, the Cox regression analysis revealed that, apart from age, other variables affect the likelihood of nursing exits. At this stage, we did not introduce these into the projection equations, either because we deemed them to be unchanging (e.g., family status) or because if they do change, we have no estimates of the degree of change. In the future, it will of course, be possible to introduce them into the projection equations according to the trends of change.

## Discussion

Israel's (RN) nursing workforce is relatively mature - 45% are above the age of 45 - and stable: most RNs work in the profession, with high survival rates even after 20 years. Furthermore, inter-organizational mobility is low, and mostly from hospital to community. The Western nursing workforce typically shows rather lengthy exits followed by re-entries [5,6,16]. The same is true of Israel, though exits are shorter and on a smaller annual scale (about 2% of the workforce at any time). Contrary to common opinion, a high percentage of RNs work full-time, particularly hospital nurses, and their weekly average of hours is high. It would thus be difficult to expand the workforce on the basis of existing manpower since most RNs are already employed; the existing workforce is being utilized nearly to the maximum.

The survival rate for working in the profession among young RNs (77% after 10 years) was found to be lower than among RNs of middle or mature age (96% after 10 years). Young RNs thereby constitute a less stable workforce. It is impossible to tell from the data whether these findings are typical of young nurses in general or apply only to the current cohort. In any case, there seems to be a similar phenomenon among young people in Israel's general workforce today: they are more mobile than were the previous cohorts (at that age). If we add the fact that the majority of the nursing workforce are women and the younger ages correspond to childbearing years, we see that the exits from the labor cycle are common, even if temporary. Since young nurses are expected to constitute half the future nursing workforce in any period, this finding has implications for the addition of new RNs.

The findings on age-linked exits from the nursing profession are consistent with those on job and professional satisfaction, which reveal that young nurses in Israel - who comprise the main reservoir of future workforce - are less satisfied with the profession than mature nurses [17]. Worldwide studies investigating the differences between young and mature nurses in this regard have revealed mixed findings [18]. According to the literature, if nurses are to be kept and maintained in the profession, a whole set of reasons and circumstances must be taken into account: financial incentives and workload reduction without impaired wages [18,19]; raising work satisfaction and developing career opportunities. There is no one solution to the problem of exits from the nursing profession [10]. Strategies adopted by employers have been varied, e.g., investing in in-house hospital training programs, improving the work environment and fostering a hospital's reputation as a good place to work [20]; encouraging professional autonomy and emphasizing personal fulfillment [21-23]. In general, apart from the above-mentioned, special efforts appear to be necessary to keep young nurses in the profession and ways should be found that are appropriate to Israel's healthcare labor market.

The finding of high cumulative survival rates among more mature RNs may affect decisions on investing efforts to recruit older candidates to the profession. More mature workforce may have a shorter working life due to late entry. On the other hand, according to the study findings, they are a particularly stable workforce who will remain in the profession (assuming that current graduates of retraining programs and the mature nurses in the study behave similarly in the labor market). The entry of relatively mature nurses into the workforce, after pursuing different occupations for several years, is viewed positively around the world today. They constitute a source of workforce to reduce the expected shortage [24], as well as a resource of knowledge and experience [18].

One variable forecasting career survival in nursing is post-basic education courses. Working RNs with higher qualifications have a higher survival rate (94% after 10 years) than those without (78% after 10 years). Further training can thus be said to contribute to workforce stability (though the claim can be made that the more "stable" personnel - i.e. those who wished to remain in the profession - are the ones who chose advance training). Higher-qualified nurses earn more and some fill administrative or specialized positions. These findings may impact decisions on the overall extent of further training. This discussion relates to the trend of specialization in the western world as advanced nursing practice: nursing that emphasizes an advanced level of performance, which maximizes the reliance on knowledge and skill in patient care [25-27].

The study findings provided a basis for more rigorous projections of the RN supply at given points in time since the equations employed rested on the characteristics of the current workforce. In the future, it will be possible to fine-tune the projection model and introduce additional variables found to affect exits, based on foreseeable trends. According to our equations, a drop of 12.5% is expected in the number of practicing RNs in five years time, and of 25.5.% - in 20 years time. In terms of nurse/population ratios, a drop from 4/1,000 RNs in 2008 to about 2/1,000 in 2028. While this model presents only supply, it appears to reinforce the claim of a future shortage of RNs. However, it would be more correct to examine projected supply against projected demand (which is related, for example, to an increase in hospital beds, changes in patient composition, in community/hospitalization ratios, in technology etc.). Thus, the study data may also serve as a basis for evidence-based decisions to predict supply versus demand in future research.

## Conclusions

This study relates to a major - certainly the largest, numerically, component of the health system: the nursing workforce. It adds another important layer to our understanding of the structure of the nursing workforce. The study findings furnish highly detailed information on the supply of RNs and the characteristics of their employment and provide more rigorous projections of supply than in the past on the declining rates of the nursing workforce in the coming decades, making it possible to improve the projections of future supply and organize future training, whether basic courses or advance training, based on evidence. The study also points to implications for policy decisions regarding the findings that the young nursing workforce is less stable (warranting special efforts to preserve it), that there are advantages to recruiting a more mature workforce, and that post-basic education is connected with workforce stability.

## Endnotes

1. The examination of the nursing distribution in the years 2000 to 2008 (the start of the study) revealed that the percentage of RNs among licensed nurses up to age 60 had already reached some 80% of all nurses in 2008, and that 61% of the practical nurses were over the age of 45. Moreover, whereas in 2000-08, an average of some 600 practical nurses annually joined the population of RNs after being retrained as such (mostly immigrant practical nurses from the Former Soviet Union [FSU]) - in 2009, this figure dropped to 380 nurses, and in 2010, to 214 nurses [14,15]. The large wave of immigration from the FSU had brought with it many nurses and then came to an end. Presumably, the practical nurses able to upgrade their status to RN would already have done so over the years. In addition, as said, since 2007, the training of practical nurses stopped. It thus follows from the above that the reservoir of practical nurses is "drying up." We assumed that in the five-year period of 2008-13, the amount of practical nurses retraining as RNs would decrease by half, to some 300 retrained nurses per year and that, subsequently (over the next five years), it would decrease still further, to some 150 per year - which is about a quarter of the average annual amount of nurses who retrained in 2000-08. In the next 10 years, the number of nurses retraining from practical nurses to RNs is expected to amount to isolated cases. This explains why we assumed that an analysis of the data on RNs would suffice for the projections of the general nursing supply in 10 and 20 years from now.

2. The term "new licensees" refers to both new RNs (duly qualified and currently entering the profession) and practical nurses already working in the system who trained further to qualify as RNs. To speak of practical nurses, then, does not signify additional new manpower in the system but a change in the number of RNs.

3. The assumption about a reduction in the number of practical nurses requalifying as RNs is mainly important for the projections of future supply in another 10 or 20 years since the projected supply for the next few years must take into account that, in addition to RNs, there are still practical nurses in the workforce.

## Abbreviations

CBS: Central Bureau of Statistics; MoH: Ministry of Health; RNs: registered nurses.

## Competing interests

The authors declare that they have no competing interests.

## Authors' contributions

NN initiated the study and was the leading researcher of this study, responsible for its design, questionnaire building, overseeing fieldwork and data analysis and drafting the manuscript. OT contributed to the initiation of the study, its design and questionnaire building. ShR and SR, both contributed to research design and questionnaire building. All authors contributed to the writing of this manuscript, and read and approved the final draft.

## Authors' information

Nurit Nirel is a senior researcher at the Smokler Center for Health Policy Research of the Myers-JDC-Brookdale Institute. She has an M.A. degree in Labor Studies from Tel Aviv University.

Shoshana Riba, RN, PhD, is the National Chief Nurse and the Director of the Israel Ministry of Health's Nursing Division. She advises the Minister of Health on all matters concerning the nursing profession and its role in the Israeli health care system and is a member of Israel's National Health Council. Dr. Riba has longstanding experience in senior management in Israel and as a consultant to nursing systems in developing countries.

Sima Reicher, RN, Ph.D., Tel Aviv University. She is the Head of Nursing Professional Guidelines Department, Nursing Division, Israel Ministry of Health. She was previously the Chief Nursing Officer of the IDF Medical Corps.

Orly Toren, RN, MSc, PhD, is a faculty member at the Hebrew University, School of Nursing. She is the assistant director of nursing for research and development at the Hadassah Medical Organization, and has served in many leading nursing managerial roles in hospitals. Her research focuses on management, nursing manpower and healthcare policy. She is one of the two editors of the first book in Hebrew on nursing management and leadership.
